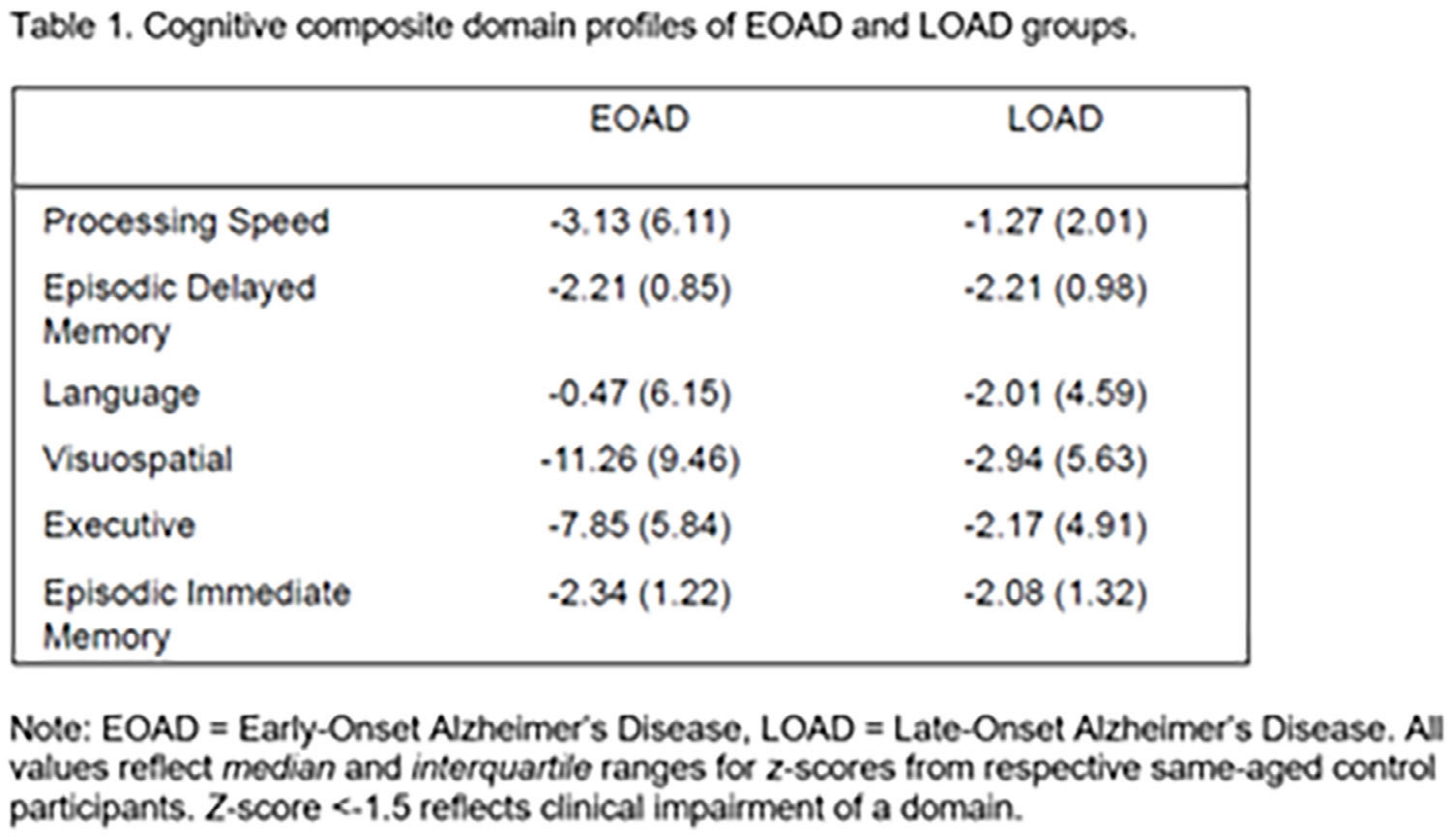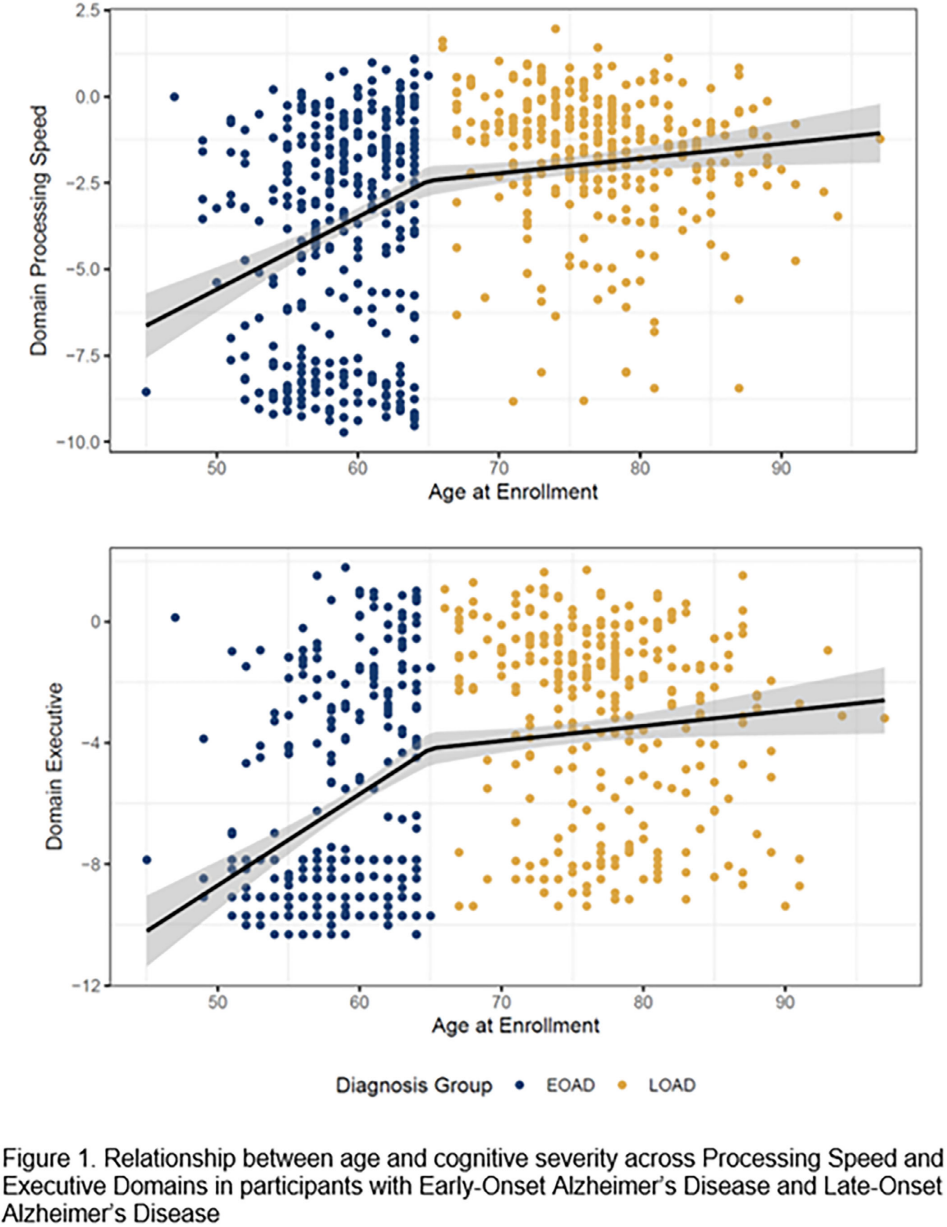# Relationship Between Age and Severity of Cognitive Impairment at Diagnosis For Early‐Onset and Late‐Onset Alzheimer’s Disease: Comparison of LEADS and ADNI

**DOI:** 10.1002/alz70861_108877

**Published:** 2025-12-23

**Authors:** Dustin B. Hammers, Ani Eloyan, Maryanne Thangarajah, Alexander Taurone, Kala Kirby, Jeffrey L. Dage, Kelly N. Nudelman, Maria C. Carrillo, Gil D. Rabinovici, Brad C. Dickerson, Liana G. Apostolova, the LEADS Consortium

**Affiliations:** ^1^ Indiana University School of Medicine, Indianapolis, IN USA; ^2^ Department of Biostatistics, Brown University, Providence, RI USA; ^3^ Brown University, Providence, RI USA; ^4^ Department of Medical and Molecular Genetics, Indiana University School of Medicine, Indianapolis, IN USA; ^5^ Department of Neurology, Indiana University School of Medicine, Indianopolis, IN USA; ^6^ Medical & Scientific Relations Division, Alzheimer's Association, Chicago, IL USA; ^7^ Memory and Aging Center, Weill Institute for Neurosciences, University of California San Francisco, San Francisco, CA USA; ^8^ Massachusetts General Hospital and Harvard Medical School, Boston, MA USA; ^9^ Department of Neurology, Indiana University School of Medicine, Indianapolis, IN USA; ^10^ Department of Radiology and Imaging Sciences, Center for Neuroimaging, Indiana University School of Medicine, Indianapolis, IN USA

## Abstract

**Background:**

Research into AD has revealed that cognitive manifestation tends to differ depending on age of onset, such that younger patients often present with worse cognitive abilities than their older counterparts until the age of 75 – with a reversal of the relationship after 75‐years‐old (Barnes et al., 2018). As recent work has identified a unique cognitive profile for Early‐Onset Alzheimer’s Disease (EOAD) relative Late‐Onset Alzheimer’s Disease (Hammers et al., 2025), it is unknown if the nature of the association between age and cognitive severity at presentation also differs across conditions.

**Method:**

Cross‐sectional baseline cognitive data were analyzed from 401 EOAD participants enrolled in the Longitudinal Early‐Onset AD Study (Apostolova et al., 2019) and 314 LOAD participants from the Alzheimer’s Disease Neuroimaging Initiative. A series of *linear spline regression* models were conducted with age at enrollment as the predictor and the specific domain composite score as the outcome variable, after accounting for sex and *APOE* ε4 status. For each model, a knot was selected at age 65, with *linear regression* applied to a total of two segments surrounding that single knot.

**Result:**

As seen in Table 1, cognitive impairments (*z*‐scores <‐1.5) were common across domains for EOAD and LOAD. Significant differences existed in the slopes of Processing Speed/Attention (*p*=.002), Executive Functioning (*p*<.001), and Episodic Immediate Memory (*p*=.007) performance between age‐segments in the model **(**Figure 1**)**. For example, performance on the domain composites for Processing Speed/Attention and Executive Functioning increased significantly for EOAD participants as the age of enrollment increased (*βs*=0.19‐0.27, *ps*<.001), whereas the performance for LOAD participants on these domains also increased – but to a lesser extent – as age increased. No age‐relationship was observed for Episodic Delayed Memory, Language, or Visuospatial performances (*p*s=.06‐.34).

**Conclusion:**

Results suggest a unique relationship between EOAD and LOAD populations for the age at enrollment and cognitive severity for executive and processing/learning domains. This supports previous findings that EOAD manifests clinically in a distinct fashion than LOAD in these domains. In the age of disease‐modifying treatments, results highlight the importance of assessing for cognitive declines in individuals starting much earlier than age 65.